# Primordial initiation, yield and yield component traits of two genotypes of oyster mushroom (*Pleurotus* spp.) as affected by various rates of lime

**DOI:** 10.1038/s41598-022-16833-9

**Published:** 2022-11-09

**Authors:** Samuel C. Chukwu, Chidiebere A. Ibeji, Chigozie Ogbu, Happiness O. Oselebe, Emmanuel O. Okporie, Mohd Y. Rafii, Yusuff Oladosu

**Affiliations:** 1 Ebonyi State University Mushroom Center, Abakaliki, Nigeria; 2 Department of Crop Production and Landscape Management, Faculty of Agriculture and Natural Resources Management, Ebonyi State University, PMB 053, Abakaliki, Nigeria; 3 Laboratory of Climate-Smart Food Crop Production, Institute of Tropical Agriculture and Food Security, Universiti Putra Malaysia, UPM Serdang, 43400 Selangor, Malaysia; 4 Department of Clinical Medicine, Ebonyi State University, Abakaliki, Nigeria; 5 Department of Crop Science, Faculty of Agriculture, Universiti Putra Malaysia, UPM Serdang, 43400 Selangor, Malaysia

**Keywords:** Biological techniques, Biotechnology, Plant sciences

## Abstract

Mushrooms are fleshy fungi valued globally for their nutritional and medical benefits. The study was conducted at Ebonyi State University Mushroom Center, Abakaliki, to determine an optimum level of limestone (CaCO_3_) on the genotypes for maximum growth and yield. The experiment was carried out as a split-plot experiment in a completely randomized design (CRD) with the use of Oyster mushroom variety. The two genotypes (GI and GII) were placed in the whole plot while limestone was placed in the sub-plot which consisted of five rates of CaCO_3_(Og,5 g, 10 g, 15 g and 20 g). Sawdust and rice husk substrates were used at the ratio of 60:40 and sterilized for six hours at 121 °C using the steam sterilization cylinder. The media bags were off–loaded after one day and allowed to further cool for another day before inoculation. The cultured spawn was used to inoculate the media upon cooling at room temperature. Data were collected on agro-morphological parameters such as primordial initiation, stalk height, stalk diameter, number of branches, number of fruits, number of productive bags, fresh and dry weights, and subjected to analysis of variance (ANOVA). The result obtained indicated that there was a significant difference (P < 0.05) between the two genotypes studied in all parameters except the dry weight of the mushroom. Also, the various rates of CaCO_3_ had a significant difference (P < 0.05) in most agro-morphological traits except stalk diameter, number of fruits and fresh weight. However, the interaction of the whole plot (genotype) and sub-plot (lime rates) showed no significant difference (P > 0.05) in all parameters evaluated except the stalk diameter. Genotype I initiated more primordial compared to primordial initiation in genotype II and they differed significantly (p < 0.05) from each other. More so, the result showed that increasing the rate of CaCO_3_ from 0 to 5 g significantly increased the primordial initiation from 17 to 22. However, further increase in lime rates above 5 g significantly reduced the primordial initiation from 22 to 15. It was concluded that the 5 g rate of limestone produced the best primordial initiation. Therefore, genotype I and 5 g of CaCO_3_ were recommended. There was also a strong relationship between the primordial initiation and most growth and yield components traits studied. There was a significant positive correlation between primordial initiation and stalk height (r = 0.799*), stalk diameter (r = 0.692*), number of mushroom branches (r = 0.773*), number of productive bags (r = 0.888*), number of fruits (r = 0.810*), fruit weight (r = 0.918*) and dry weight (r = 0.916*). Ideal conditions that would guarantee more primordial initiation for higher yield were recommended.

## Introduction

Mushrooms (*Pleurotus* spp.) are valued globally for their nutritional and medicinal benefits.They contain little proportion of fats and the fatty acid fractions are mainly in the unsaturated forms like linoleic acid. Mushrooms provide excellent nutrition for ensuring the proper condition of the heart, as well as the cardiovascular system^1^. Mushroom consumption was initially confined to some regions of the world, but technology and civilization have made it possible for the consumption of mushroom to spread to various regions. Recently, mushroom consumption has gained more acceptability in different areas and is utilized in preparing various dishes. There are also great export opportunities created by mushroom as different countries utilize them for various benefits. Mushroom could be consumed in various forms ranging from fresh, dried, pickled, canned, powdered, etc^2^. Due to the low cost of input with expected high returns, more farmers and entrepreneurs have ventured into mushroom production.

Mushrooms are edible fleshy fungi, belonging to the class *Basidiomycota* and order *Agaricomycetes.* They have a stem bearing a cap with gills under the cap^3^. Although they are edible, some mushrooms could be wild and toxic, i.e., non-edible. Mushrooms are made up of over 90% of water and fat content is below 1%. It also contains vitamin B and other elements such as selenium and copper with little quantity of sodium. Mushrooms are unique compared to other vegetables and food products because of the natural embodiment of vitamin D which could alternatively be sourced from animals and poultry. Vegetables and other food products such as milk are usually fortified with vitamin D but such fortification is not required in mushroom. Mushrooms are usually seen as saprophytes on wood, soil and farmlands, open fields and roadsides. The large fruiting bodies are very obvious to observe using un-aided eyes. Mushroom has various tastes, texture and flavor. Fresh mushroom contains about 80–95% moisture, 0.3–0.4% fat and 1% minerals and vitamins. Mushrooms are not destroyed by cooking, drying and freezing^4,5^. Edible mushrooms are recommended by the Food and Agriculture Organization (FAO) as a source of protein for developing countries that feed mostly on cereal crops. Oyster mushroom, straw and ear mushrooms are the three types of mushrooms currently under commercial cultivation worldwide, especially in Africa, Asia and Europe. Cultivation of mushrooms could be done both under temperate and tropical climate conditions all year round. The *Pleurotus* species are ranked third after white button and shiitake in world production^6^. Mushrooms are known for bio-conversion of crop and plant residues from forest and agriculture to protein, vitamins and minerals^5,7^.

Large-scale mushroom producers have an advantage over small scale farmers due to access to technology and capital, hence they produce large quantities of quality mushroom to meet up with the market demand^8^. In order to overcome the challenges facing small-scale farmers, developing countries are encouraging collective action through farmer’s cooperative societies to improve their access to technology and credit facilities^9–11^. Forest contributes to all aspects of rural life, providing food, fodder, fuel, building materials and household items. Mushrooms are used as food and it has greater importance in human diets worldwide than ever before. There is a higher mineral content in mushroom compared to meat, fish and most vegetables^12^. Deforestation, bush burning and over-exploitation of forest products should be discouraged to reduce the threat to the availability of mushroom in Nigeria. The National Mushroom Development Project introduced in 1990 for the production of exotic mushrooms helped the urban unemployed youths to establish small-scale mushroom farms while technologies were used for straw mushroom production^13^.

The growth promoting substances available in the mushroom substrates depends on the pH range. The growth and development of mushrooms are affected by the pH of the substrate. Because pH affects nutrition and morphological features of mushroom, the pH of the substrate should be slightly acidic to slightly basic, i.e., near neutral. According to Faryal et al.^14^, the optimum pH range for mycelia growth and primordial initiation is 5.5–6.5. Also, Bellettini et al.^15^ noted that various strains of *Pleurotus* species required different optimum pH ranges. Even though mushroom can thrive in varied temperature conditions, the Mycelium of fungi (mushroom) obtain nutrients from substrate and performs better at the specific level of pH^16^. Lime is utilized in mushroom production to moderate the pH of the substrate. The rapid growth of mushroom mycelia (*Pleurotus sajor-caju*) takes place at 6.4–7.8 pH^17^. Lee et al.^18^ on using CaCO_3_ as a liming material reported that the oyster mushroom performed best at pH near neutral and slightly basic. Asneti^19^also recorded superior performance of mushroom at 2% lime when produced using wet wheat straw as a medium, while Maurya et al.^20^ reported that the best quality spawn of oyster mushroom was produced on pearl millet and sorghum with a 1:1 lime: gypsum ratio. The objective of the study was to adjust the limestone concentration of substrates to determine the optimum level of CaCO_3_ for maximum yield of the two genotypes of mushroom.

## Result

### Agro-morphological traits

The result in Table 1 shows that genotype I had more primordial initiation compared to genotype II and they differed significantly (P < 0.05) from each other. Limestone levels had a significant difference (P < 0.05) in the number of primordial initiation. Primordial initiation increased as lime rates increased from 0 to 5 g. After this rate, primordial initiation began to decrease with a further increase in lime rates from 10 to 20 g.The interaction effect of the oyster mushroom varieties and limestone levels was not significant (Table 1). The highest number of primordial initiation was from genotype I and 5 g of lime (CaCO_3_) whereas the least number of primordial initiation was in genotype II and 20 g concentration of lime.

**Table 1:** Effects of genotypes and limestone levels on growth attributes of the mushroom.

Levels	Primordial initiation			Stalk height			Stalk diameter			No. of branches		
	GI	GII	Mean	GI	GII	Mean	GI	GII	Mean	GI	GII	Mean
0 g	18.67	15.67	17.17bc	6.27	6.83	6.55a	1.84	1.93	1.89a	14.00	10.72	12.36b
5 g	26.33	17.33	21.83a	8.90	5.67	7.29a	2.25	1.41	1.83a	19.67	15.78	17.72a
10 g	24.33	17.67	21.00ab	8.20	6.43	7.32a	2.41	1.56	1.99a	13.50	12.50	12.90b
15 g	21.33	14.00	17.65abc	8.57	5.67	7.12a	2.35	1.57	1.96a	11.30	7.57	9.43c
20 g	17.67	11.67	14.67c	8.53	5.50	7.02a	2.50	1.45	1.98a	7.90	6.57	7.23c
Mean	21.67a	15.27b		8.09a	6.03b		2.27a	1.90b		13.27a	10.63b	
CV	16.94	16.29		12.96	9.61		11.32	12.97		32.46	35.17	

Means with the same letter are not significantly different at P = 0.05.

LSD_0.05_ for comparing two spawn genotypes means = 2.735.

LSD_0.05_ for comparing two lime means = 4.325.

LSD_0.05_ for comparing two spawn genotypes means = 1.960.

LSD_0.05_ for comparing two lime means = 3.098.

LSD_0.05_ for comparing two spawn genotypes means = 0.553.

LSD_0.05_ for comparing two lime means = 0.875.

LSD_0.05_ for comparing two spawn genotypes means = 1.708.

LSD_0.05_ for comparing two lime means = 2.700.

The two oyster mushroom genotypes had a difference significant (P < 0.05) in stalk height. Taller mushrooms were obtained from genotype 1 compared to genotype II. The effect of the limestone (CaCO_3_) on the height of the stalk was also significantly different (P < 0.05) (Table 1). The result indicated that 5 g of CaCO_3_ added to the substrates recorded the highest stalk height and this result was similar to 10 g, 5 g and 10 g of limestone (CaCO_3_) rates and was significantly taller than oyster mushrooms in substrate bags with 15 g, 20 g and 0 g limestone rates.

Results showed that there was a significant difference (P < 0.05) between the two mushroom genotypes studied. The stalk diameter of genotype I was greater than that of genotype II and they differed significantly (P < 0.05). Limestone levels had no significant difference (P > 0.05) in the stalk diameter of the oyster mushrooms. The treatment combination of the two mushroom genotypes and limestone rates interaction showed a significant effect (Table 1).

The highest number of mushroom branches was recorded in genotype 1 (Table 1) which differed significantly (P < 0.05) from genotype II. The levels of limestone (CaCO_3_) had a significant difference (P < 0.05) in the number of branches. As shown in Table 1, 5 g of limestone (CaCO_3_) produced mushrooms with the highest number of branches.10 g of limestone also produced a similar result and the least number of branches was produced from 15 g, 20 g and 0 g. The results showed that 5 g limestone rate differed significantly from 20 g, 15 g, 10 g and 0 g (P < 0.05) while 10 g limestone rate differed significantly from 20 and 15 g but was the same as the 0 g (P < 0.05). 0 g limestone rate differed significantly from 20 and 15 g (P < 0.05).The treatment combination of the two genotypes and limestone levels had no significant difference (Table 1). Nevertheless, genotype 1 and 5 g of limestone gave the highest number of branches.

### Yield and yield component traits

The result presented in Table 2 on the effect of mushroom genotypes on the number of productive bags was found to have a significant difference (P < 0.05). However, genotype I recorded the highest number of productive bags which differed significantly from the number of productive bags recorded in genotype II. The effect of limestone (CaCO_3_) levels as shown in (Table 2) on the number of the productive bag was significantly different (P < 0.05). 5 g and 10 g of limestone gave the highest number of productive bags compared to the results from 15 g, 20 g, and 0 g which produced a lesser number of productive bags. The least number of productive bags was found in 20 g limestone concentration. The results showed that 5 g limestone (CaCO_3_) rate was significantly different from 20 and 15 g but statistically the same as 0 g and 10 g rates (P > 0.05). Also, 10 g of CaCO_3_ was significantly different (P < 0.05) from 20 and 15 g but significantly similar to 0 g and 5 g (P > 0.05). The interaction effect of the varieties of oyster mushroom and limestone levels on the number of productive bags of oyster mushroom was non-significant (P > 0.05). However, the highest number of productive bags was obtained from genotype I and 5 g of limestone, while the least was obtained from genotype II and 20 g of limestone.

**Table 2:** Effects of genotypes and limestone levels on yield and yield component traits of the mushroom.

Levels	Productive bags			Unproductive bags			No. of fruits			Fresh weight			Dry weight		
	GI	GII	Mean	GI	GII	Mean	GI	GII	Mean	GI	GII	Mean	GI	GII	Mean
0 g	5.30	4.67	4.99ab	0.67	1.33	1.00ab	11.30	7.30	9.30a	37.40	35.06	36.23a	20.67	12.67	16.67c
5 g	6.00	5.30	5.65a	0.00	1.00	0.50b	14.67	4.00	9.34a	54.76	28.11	41.44a	29.33	22.67	26.00a
10 g	6.00	5.00	5.50a	0.00	1.00	0.50b	14.67	6.00	10.34a	55.72	36.05	45.89a	26.33	16.67	21.50b
15 g	5.00	3.67	4.33bc	1.00	2.33	1.64ab	12.00	5.33	8.67a	47.16	26.84	37.00a	19.00	10.00	14.50cd
20 g	4.67	2.67	3.67c	1.00	3.33	2.16a	13.30	5.33	9.32a	47.21	19.61	33.41a	14.00	9.00	11.50d
Mean	5.39a	4.26b		0.53a	1.80b		13.19a	5.59b		48.45a	29.13b		21.87a	14.20b	
CV	11.06	25.36		94.71	56.46		11.61	21.45		15.24	23.02		27.74	39.34	

Means with the same letter are not significantly different at P = 0.05.

LSD_0.05_ for comparing two spawn genotypes means = 0.694.

LSD_0.05_ for comparing two lime means = 1.097.

LSD_0.05_ for comparing two spawn genotypes means = 0.796.

LSD_0.05_ for comparing two lime means = 1.259.

LSD_0.05_ for comparing two spawn genotypes means = 3.075.

LSD_0.05_ for comparing two lime means = 4.862.

LSD_0.05_ for comparing two spawn genotypes means = 12.595.

LSD_0.05_ for comparing two lime means = 19.918.

LSD_0.05_ for comparing two spawn genotypes means = 2.322.

LSD_0.05_ for comparing two lime means = 3.671.

The effect of the oyster mushroom genotypes on the number of unproductive bags of oyster mushroom in (Table 2) was significant (P < 0.05). Genotype 1 gave the least number of unproductive bags while the highest value was recorded in genotype II and they differed significantly (P < 0.05). Limestone levels were significant in the resulting number of unproductive bags of oyster mushroom. Two rates; 5 g and 10 g of limestone (CaCO_3_) gave the least number of unproductive bags while the greater number of unproductive bags was obtained from 15 g, 20 g and 0 g of limestone. The treatment combination of the oyster mushroom genotypes and limestone levels on unproductive bags of oyster mushroom was non-significant (P > 0.05). Though, the highest number of unproductive bags was recorded in genotype II and 20 g of limestone while the least number was obtained from genotype 1 and 5 g of limestone. The results indicated that 20 g level of limestone differed significantly from 10 g, 5 g and 0 g but the same as 15 g. Also, 15 g limestone level differed significantly from 10 and 5 g levels of CaCO_3_ but was the same as 0 g limestone level (P > 0.05).

Results revealed that mushroom genotypes had a significant effect on the number of fruits. The highest number (13.19) of fruits was recorded in genotype I while genotype II produced the least (5.59), and they differed significantly (P < 0.05) from each other. There was no significant difference in limestone rates, as well as interaction effects (Table 2).The two varieties of oyster mushroom had a significant effect on the fresh weight of the mushroom (Table 2). The highest fresh weight was recorded in genotype I and the least in genotype II and they differed significantly (P < 0.05). The effect of limestone levels (CaCO_3_) on the fresh weight of mushroom had no significant difference. A non-significant difference was also observed in the interaction results (Table 2).

As presented in Table 2, there was a significant difference (P < 0.05) between the two oyster mushroom genotypes on the dry weight. The highest dry weight value was recorded in genotype I while the lowest was recorded in genotype II and they differed significantly (P < 0.05). The effect of limestone (CaCO_3_) rates on the dry weight also had a significant difference (P < 0.05). However, 5 g and 10 g limestone concentrations produced the highest dry weight. Results showed that 5 g limestone rate differed significantly from all other limestone rates studied (0 g, 15 g, 10 g, and 20 g). Also, 0 g limestone rate differed significantly from 20 g lime rate was the same as 15 g. There was no significant difference in the interaction effects (Table 2).

### Relationship among the traits studied

The result in Table 3 shows that there was a significant positive correlation between primordial initiation and stalk height (r = 0.799*), stalk diameter (r = 0.692*), number of mushroom branches (r = 0.773*), number of productive bags (r = 0.888*), number of fruits (r = 0.810*), fruit weight (r = 0.918*) and dry weight (r = 0.916*). However, primordial initiation correlated negatively with number of unproductive bags (r = -0.897*). This result indicates that increasing the primordial initiation would significantly increase other traits studied except number of unproductive bags. Positive and significant correlations were also observed between stalk height and stalk diameter (r = 0.948*), number of fruits (r = 0.897*) and fruit weight (r = 0.937*). Stalk diameter also had a positive and significant correlation with number of fruits (r = 0.927*) and fruit weight (r = 0.902*). The number of mushroom branches correlated positive and significantly with the number of productive bags (r = 0.840*) and dry weight (r = 0.919*). The number of productive bags also had a positive and significant correlation with fruit weight and dry weight with r = 0.794* and 0.907*, respectively. Unproductive bags had negative correlations with all other traits indicating that a reduction in the number of unproductive bags could lead to an increase in other traits studied, and vice-versa. In addition, the number of fruits correlated positively and significantly with fruit weight (r = 0.927*) while fruit weight had a positive and significant relationship with dry weight (r = 0.716*) (Table 3).

**Table 3:** Correlation coefficients between primordial initiation and other agro-morphological traits studied.

Pearson correlation coefficients, N = 10 Prob >|r| under H0: Rho = 0									
	PI	SH	SD	NB	NPB	NUPB	NF	FW	DW
PI	1.000								
SH	0.799**	1.000							
SD	0.692*	0.948**	1.000						
NB	0.773**	0.335^ns^	0.157^ns^	1.000					
NPB	0.888**	0.597^ns^	0.522^ns^	0.840**	1.000				
NUPB	−0.897**	−0.674*	−0.616^ns^	−0.782**	−0.989**	1.000			
NF	0.810**	0.897**	0.927**	0.359^ns^	0.616^ns^	−0.701*	1.000		
FW	0.918**	0.937**	0.902**	0.507^ns^	0.794^ns^	−0.855**	0.927**	1.000	
DW	0.916**	0.536^ns^	0.416^ns^	0.919**	0.907**	−0.872**	0.601^ns^	0.716*	1.000

*PI* primordial initiation, *SH* stalk height, *SD* stalk diameter, *NB* number of branches, *NPB* number of productive bags, *NUPB* number of unproductive bags, *NF* number of flowers, *FW* fresh weight, *DW* dry weight.

*Significant at P = 0.05, **significant at P = 0.01, ^ns^ not significant.

## Discussion

Primordial initiation is one of the main parts of the emergence of mushrooms. Results obtained from this study showed that primordial initiation was faster in treatments having genotype 1 and 5 g of lime. It was deduced from the result that primordial initiation increased from 18.67 to 26.33 as lime rate increased from 0 to 5 g, respectively. The primordial initiation declined as lime rate was further increased to 10 g, 15 g and 20 g with pin head settings of 24.33, 21.33 and 17.67, respectively (Table 1). Primordial initiation is the growth of tiny mushroom fruiting bodies with a size of about 0.01 mm. They start developing soon after the completion of spawn running. Adjusting the pH by varying the rate of lime added to the substrate could directly affect the primordial initiation. Khan et al.^21^ found that various rates of lime had a significant difference in the number of days taken for primordial initiation. The result revealed that 0% lime and 2% lime both facilitated primordial initiation three days after completing spawn running while the further increase of lime to 4% and 6% significantly delayed primordial initiation to five and six days after spawn running, respectively. The study conducted by Hlerema et al.^22^ on mushroom production using maize industry’s waste materials showed that spawn running lasted 17–20 days while primordial initiation started one week after the completion of spawn running. This result corresponds with the findings made by Khan et al.^21^and Tirkey et al.^23^ on the number of pinheads (primordial initiation). The result showed that there was an increase in primordial initiation from 0% (28.3) to 2% (30.8) with a significant reduction in primordial initiation as lime rate increased from 4% (20.4) to 6% (11.8). Also, Khan et al.^21^and Bhattacharjya et al.^24^ studied oyster mushroom production on various lingo cellulosic substrates and observed that primordial initiation took 7–8 days after spawn running while the formation of sporocarps occurred after 10–12 days after mycelial growth.

The mushroom branches are also known as the fruiting bodies or the sporophore. Good environmental conditions of the media such as high relative humidity and low temperature enhance the production of healthy mushroom fruiting bodies. Most species of oyster mushroom require relative humidity above 85% and temperature between 15 and 25 °C for maximum growth and yield^25,26^. The result of this present study agrees with Khan et al.^21^ who found that 6% lime with pH 8.7 produced a minimum number of mushroom fruiting bodies (7.4) followed by 4% lime (16.4), 0% lime (24.2) and 2% lime (26.8) at pH 8.2, 7.2 and 7.8, respectively. The least lime rate applied, i.e., 2% produced the highest number of fruiting bodies (26.8) which differed significantly from the number of fruiting bodies recorded in 0%, 4% and 6%. The result is also in agreement with Mondal et al.^27^ who worked on various substrate compositions for oyster mushroom cultivation. Spawn running was under a steady temperature of 25 °C while fruiting was at a temperature range of 17–22 °C. They also found that spawn running took two to three weeks upon inoculation while primordial initiation started after 6–7 weeks after spawn running was completed. Fruiting bodies developed after 3 to 6 days of primordial initiation and took 27–34 days after inoculation of spawn. They also reported that 100% sawdust produced the highest yield of 646.9 g, the production efficiency of 64.69% and the number of fruiting bodies put at 22.11^28^.

**Figure 1:**
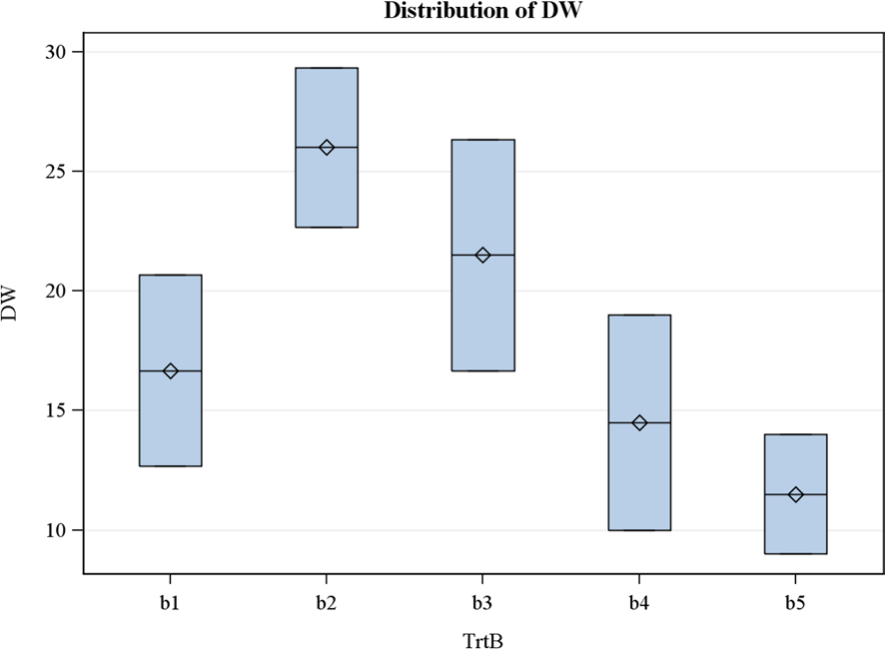
Effects of limestone levels on the dry weight of mushroom.

The result shows that increasing the lime dosage beyond 5 g led to a decline in the yield of the oyster mushroom (Fig. 1). Genotype I also produced a higher yield compared to genotype II (Table 2). The result obtained on the yield of mushroom from this study agrees with that of Bedford and Rousseau^29^ who reported that mushroom yield increased from 53.08 to 56.32 g with an increase in lime from 0 to 2%, respectively. However, the yield of mushroom significantly decreased to 32.17–23.37 g with a further increase in lime rate to 4–6%, respectively. Substrates composed of 2% lime and that of control (0% lime) had the same significance level at pH 7.8 and 7.2 with the mushroom fresh weight of 56.32 g and 53.08 g, respectively but differed significantly from substrates composed of 4% lime and 6% lime with pH of 8.2 and 8.7, respectively. Other researchers have also reported similar results. Liaqat et al.^30^ worked on the response of various means of preparing compost and limestone concentration on the yield and yield components of *Pleurotus sajor-caju* and reported that wet wheat straw and 2% of CaCO_3_ produced the fastest spawn running (23 days), early primordial initiation (48 days), the highest number of flushes (six) and the highest mushroom yield (295 g/1.5 kg substrate). Significant positive correlation between primordial initiation and other growth and yield attributes observed in this study (Table 3) corresponds with the findings made by Chukwu et al.^31^. Selecting for high primordial initiation would lead to an increase in the yield of the oyster mushroom.

## Conclusion

The treatment combination comprising genotype I and 5 g of lime showed the best results in primordial initiation and other growth and yield component traits of the oyster mushroom which included stalk height, stalk diameter and number of mushroom branches, number of fruits, fresh weight, dry weight and productive bags. Although lime was necessary for adjusting the pH of the substrates, the maximum concentration of 5 g was ideal for maximum yield. Higher concentrations above 5 g are discouraged, as such led to a decline in the yield of the oyster mushroom. It can also be concluded that there was a strong relationship between the primordial initiation and other growth and yield components traits studied. Ideal conditions that would guarantee the initiation of more primordial (pin heads) for higher yield are recommended.

## Materials and method

This experiment was carried out at the Ebonyi State University Mushroom Centre, Abakaliki, from February to November 2021 under a homogeneous environment with high relative humidity. The Center lies at the latitude 06°40′N and longitude 08°65′E and an altitude of 91.44 m above sea level. This area is characterized by a bimodal pattern of rainfall (April–July) and (September–November) with a short dry spell in August normally called the “August Break”, The total rainfall ranges from 1700 to 2000mm^32^.

### Experimental design

**Figure 2:**
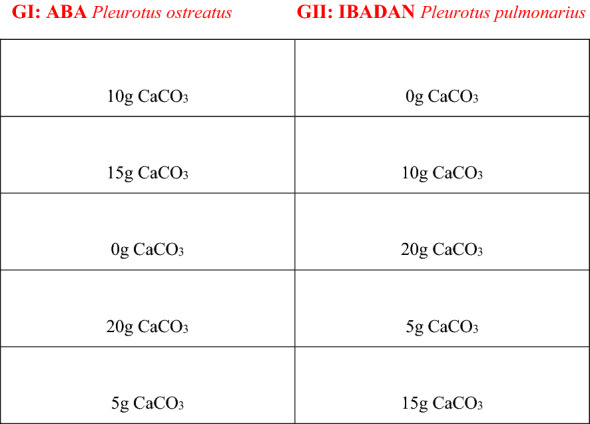
The split plot experiment design.

The study was conducted as a split-plot experiment laid out in a completely randomized design (CRD). The whole plot comprised two spawn genotypes (GI: ABA—*Pleurotus ostreatus* and GII: IBADAN—*Pleurotus pulmonarius*) while the sub-plots consists of five rates of CaCO_3_ (0 g, 5 g, 10 g, 15 g, 20 g) and the ten treatment combinations were replicated three times. The arrangement of treatments (experiment design) is shown in Fig. 2.

### Isolation and tissue culture

Samples were isolated from the desired fruiting bodies (basidiocarps) of mushroom from which pure cultures were obtained. The uncontaminated tissues were obtained by pulling apart the cap of the mushroom. The isolated samples were cut into tiny pieces with the aid of sterile fine-tip tweezers and surface-sterilized using Sodium Hypochlorite solution and washed off in distilled water before transferring to the potato dextrose agar (PDA) plate. Sub-culturing was done under aseptic conditions using the laminar flow hood to avoid any form of contamination. New cultures were re-labeled and kept as pure/mother cultures. The pure cultures were stored at 4 °C (39 °F)^33^.

### Mother spawn preparation

The millet seeds were soaked for about 12 h. The seeds were drained and transferred into 1000 ml conical flask. The seeds were sterilized inside the flask using an autoclave to ensure there was no contamination. The flasks were half-filled with the millet seeds to create enough space for mycelia growth. The seeds were shaken vigorously soon after it was brought out of the autoclave to mix the seeds on the top part of the flask, which tend to be drier compared to the seeds at the bottom of the flask where moisture drops while cooling. Preventing agglutination of the seeds into a single big mass could be another reason for shaking thoroughly^34^.

### Media preparation and sterilization

After the individual collection of substrates, they were weighed according to the ratio of 80:20 kg of sawdust and rice husk, respectively. The substrate compositions were mixed with CaCO_3_ (Lime) at five different levels as described earlier in the experiment design. All the media was sterilized using heat from steam at 121 °C for 8 h using the steam sterilization cylinder. Then, all media was left to cool for 24 h before off-loading from the cylinder and further allowed to cool for another 24 h at room temperature before inoculation^35^.

### Inoculation, spawn running and fruiting

After sterilization, the inoculation room was fumigated. The media was carried to the inoculation room. In the inoculation room, each of the media was inoculated with 10 g of spawn under aseptic conditions. Proper distribution of the spawn on top of the media was ensured. During inoculation, temperature was maintained at 18 °C, relative humidity 65 °C. The substrates were transferred inside the incubation room with ambient temperature maintained and relative humidity of 80–85% for 5 weeks. A watering-can was used to sprinkle water on the media, twice daily in the early hours of the day and the evening. At the end of the colonization period, the bags were taken to the fruiting room and rearranged horizontally. After the mycelium is fully colonized and the media are completely white, the upper part of the bags was unfolded to induce fruiting for the first flush. The pin head formation could be seen on the surface of the media after two to three days. The pinheads of the mushrooms grow into full size within three to five days intervals after pinhead formation^34,36^.

### Data collection and analysis

Data were collected and properly recorded on primordial initiation, stalk height and diameter, number of mushroom branches, number of productive and unproductive bags, number of fruits, fresh weight and dry weight of mushroom. Data collected were subjected to statistical analysis of variance (ANOVA) for split-plot experiment in completely randomized design (CRD) using SAS software version 9.4. Separation of treatment means for significant effect and correlation analysis were also done using the SAS software^37^.

## Data Availability

The datasets used and/or analysed during the current study available from the corresponding author on reasonable request. Such request should be directed to chukwu.samuel@ebsu.edu.ng.
